# Supplementary material for: High Energy Density in All-Organic Polyimide-Based Composite Film by Doping of Polyvinylidene Fluoride-Based Relaxor Ferroelectrics
Source: Polymers (Basel). 2024 Apr 18;16(8):1138. doi: 10.3390/polym16081138 (PMC11054177; doi:10.3390/polym16081138)
Supplement: Supplementary file 1 [file polymers-16-01138-s001.zip › polymers-2921335-supplementary.pdf]

Article

High Energy Density in All-Organic Polyimide-Based Composite Film by Doping of Polyvinylidene Fluoride-Based Relaxor Ferroelectrics

Chengwei Wang [1], Yue Shen [2], Xiaodan Cao [2], Xin Zheng [2] and Kailiang Ren [1,2,3,*]

1 Beijing Institute of Nanoenergy and Nanosystems, Chinese Academy of Sciences, Beijing 100083, China; wangchengwei@binn.cas.cn
2 Center on Nano Energy Research, School of Physical Science and Technology, Guangxi University,
Nanning 530004, China; shenyue08091998@163.com (Y.S.); caoxiaodan@binn.cas.cn (X.C.); zhengxin9911@gmail.com (X.Z.)
3 School of Nanoscience and Engineering, University of Chinese Academy of Sciences, Beijing 100049, China;
* Correspondence: renkailiang@binn.cas.cn

Figure S1. (a) Scanning electron microscopy (SEM) images of cross-section of Terp/PI-5; and energy dispersive spectroscopy (EDS) images of Terp/PI-5 of (b) N, (c) F, and (d) O elements. Actual optical images of (e_1) pristine PI, (e_2) Terp/PI-1, (e_3) Terp/PI-5, (e_4) Terp/PI-10 films.

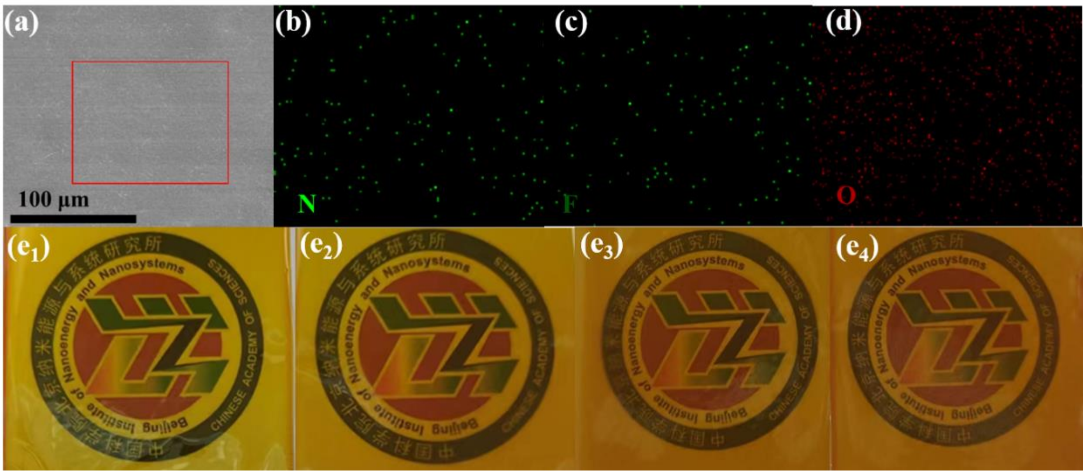

Figure S2. SEM images of (a) PI and Terp/PI composite films with different terpolymer contents (b) Terp/PI-1, (c) Terp/PI-5, (d) Terp/PI-10.

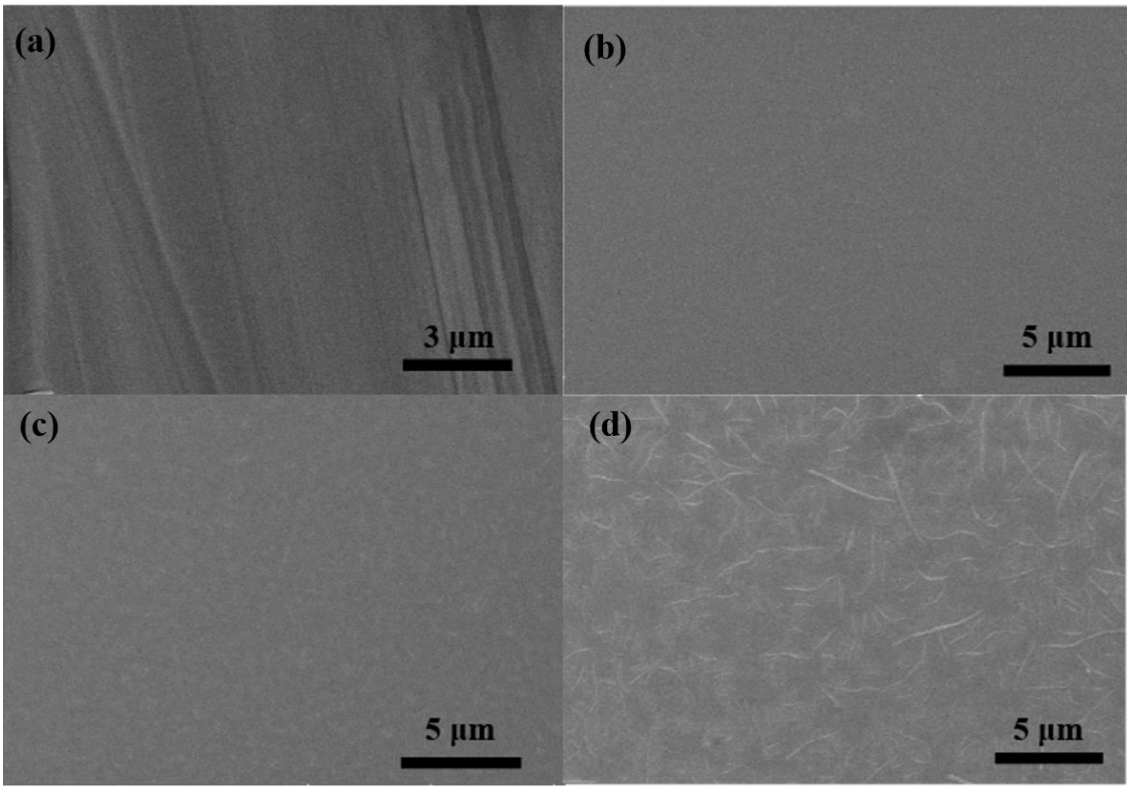

Figure S3. FTIR spectra of (a) P(VDF-TrFE-CFE) film, (b) Terp/PI composite films with various terpolymer contents.

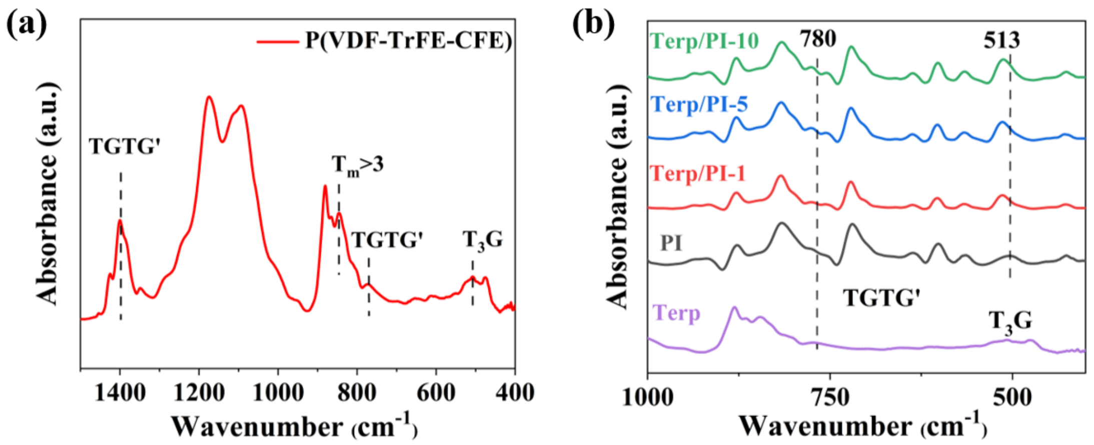

Figure S4. (a) Thermal gravimetric analysis (TGA) and differential scanning calorimetry (DSC) curves of P(VDF-TrFE-CFE), (b) TGA curves of Terp/PI composite films with various terpolymer contents.

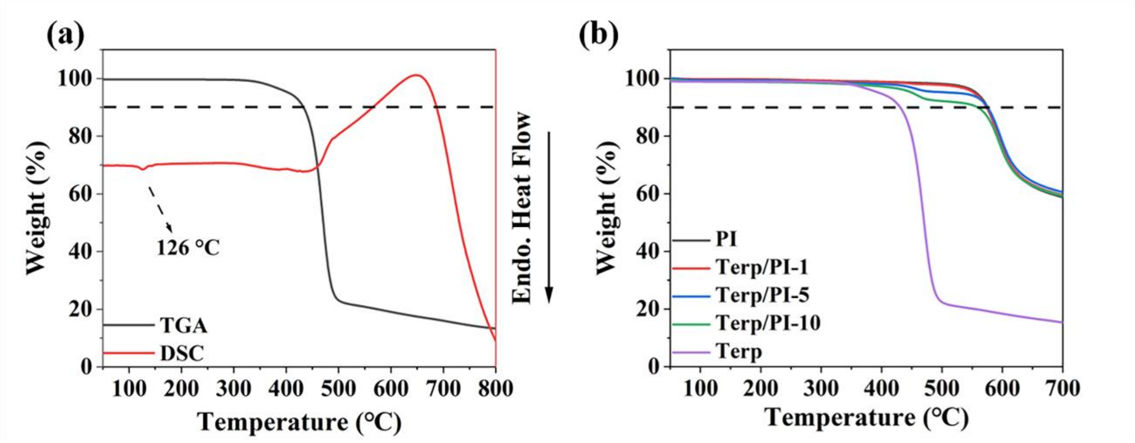

Figure S5. Dielectric constant and dielectric loss of P(VDF-TrFE-CFE) terpolymer film.

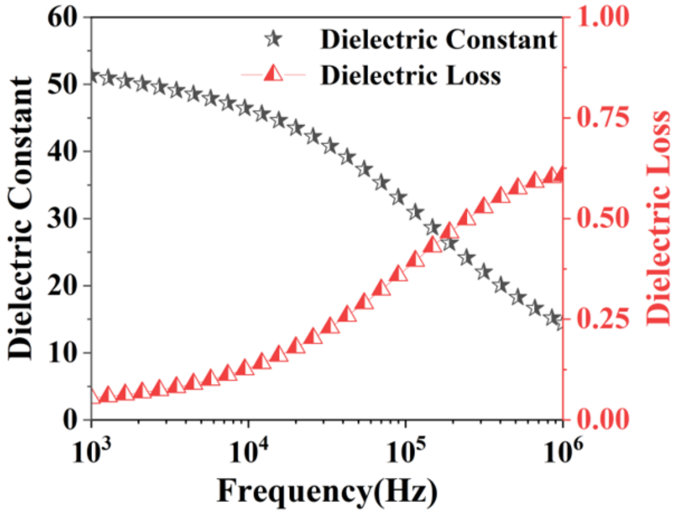

Figure S6. P-E loop at room temperature of (a) pristine PI, (b) Terp/PI-1, (c) Terp/PI-5, (d) Terp/PI-10 films.

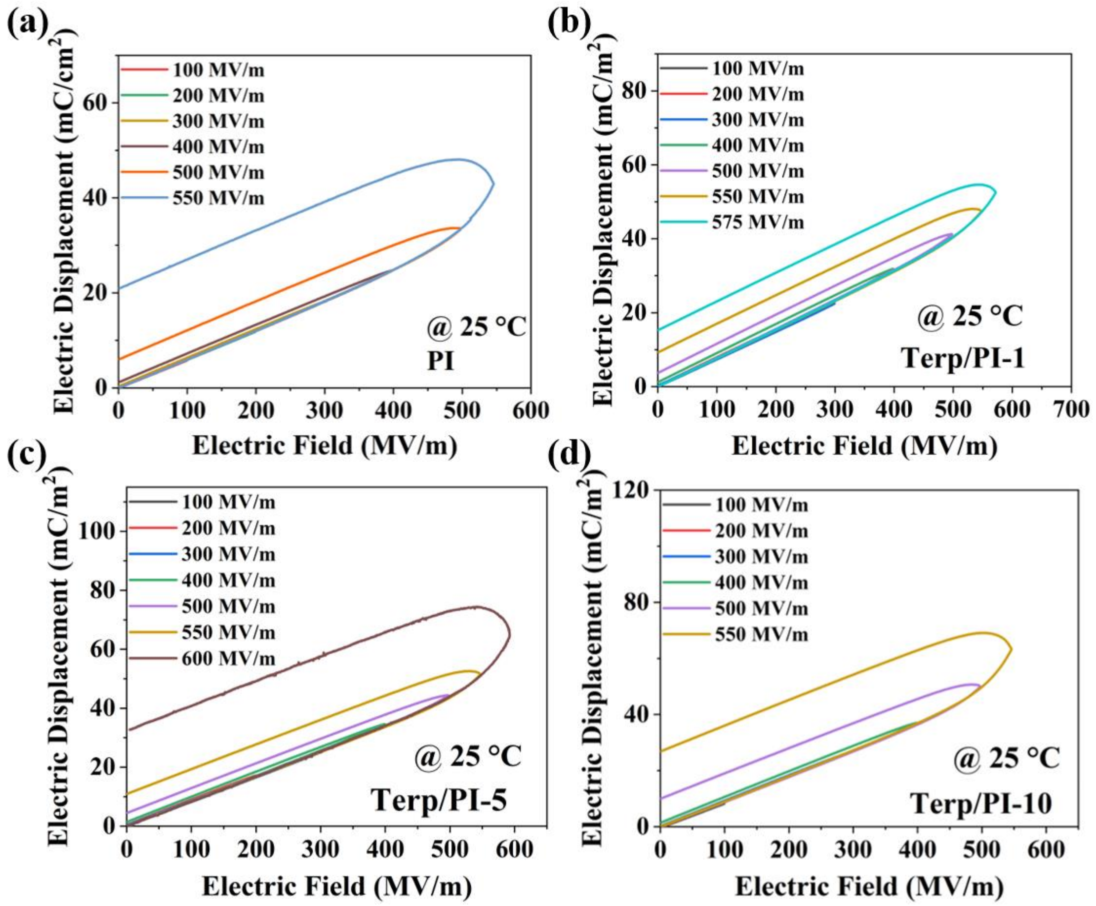

Figure S7. P-E loops data measured at 150 °C of (a) pristine PI, (b) Terp/PI-1,(c) Terp/PI-5, (d) Terp/PI-10 films.

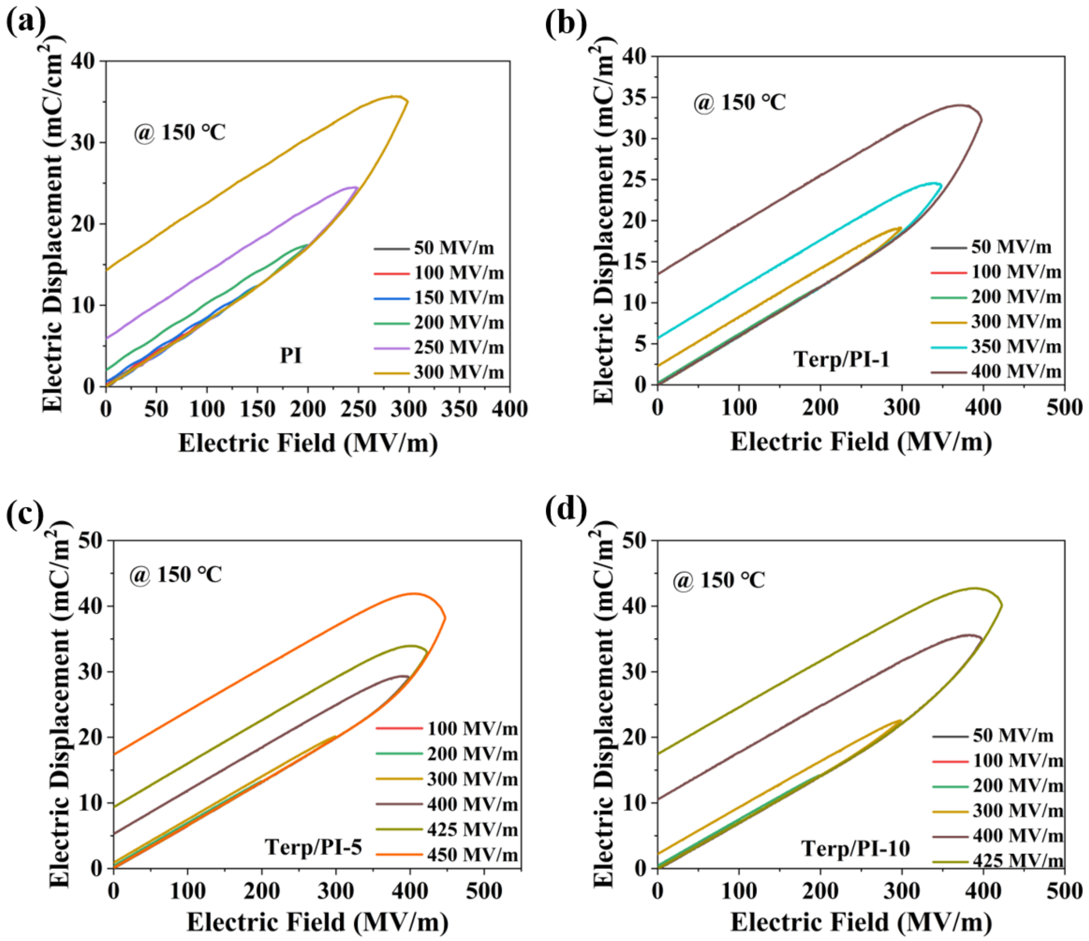

Figure S8. Fitting results for the current density as a function of electric field for the pristine PI and Terp/PI films at 150 °C using Schottky emission model.

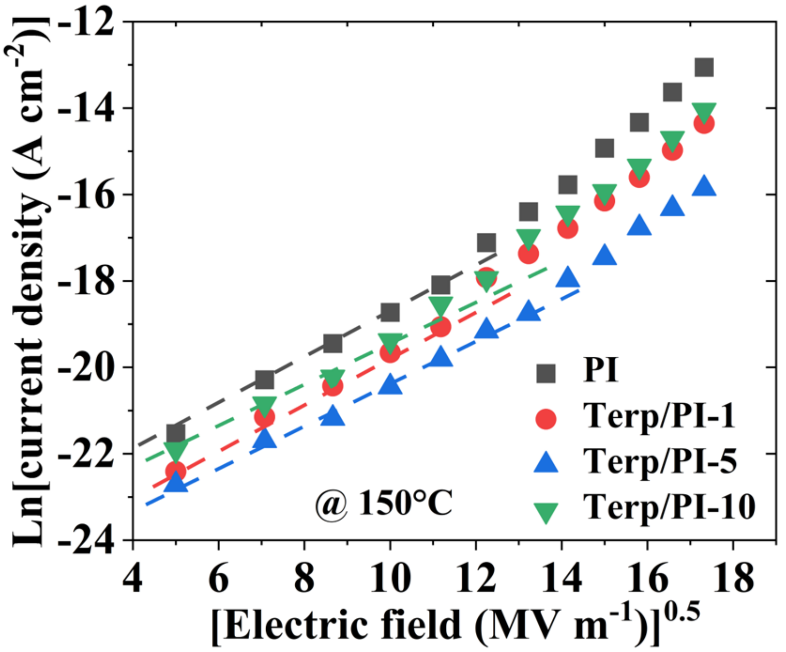

Figure S9. Statistical results for the electrostatic potential distribution on the molecular surface of (a) pristine PI and (b) P(VDF-TrFE-CFE) terpolymer.

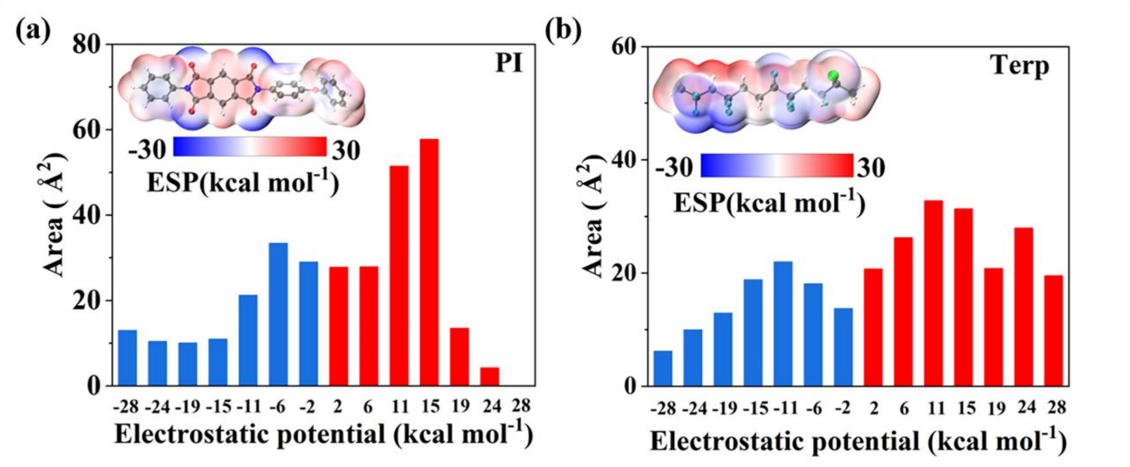

Figure S10. (a) Ultraviolet-visible light-near infrared (UV-Vis-NIR), (b) plot of $(\alpha h\nu)^{0.5}$ vs. hν data acquired from the UV-Vis-NIR spectra, (c) and (d) Binding energy obtained from ultraviolet photoelectron spectroscopy (UPS) of pristine PI and P(VDF-TrFE-CFE).

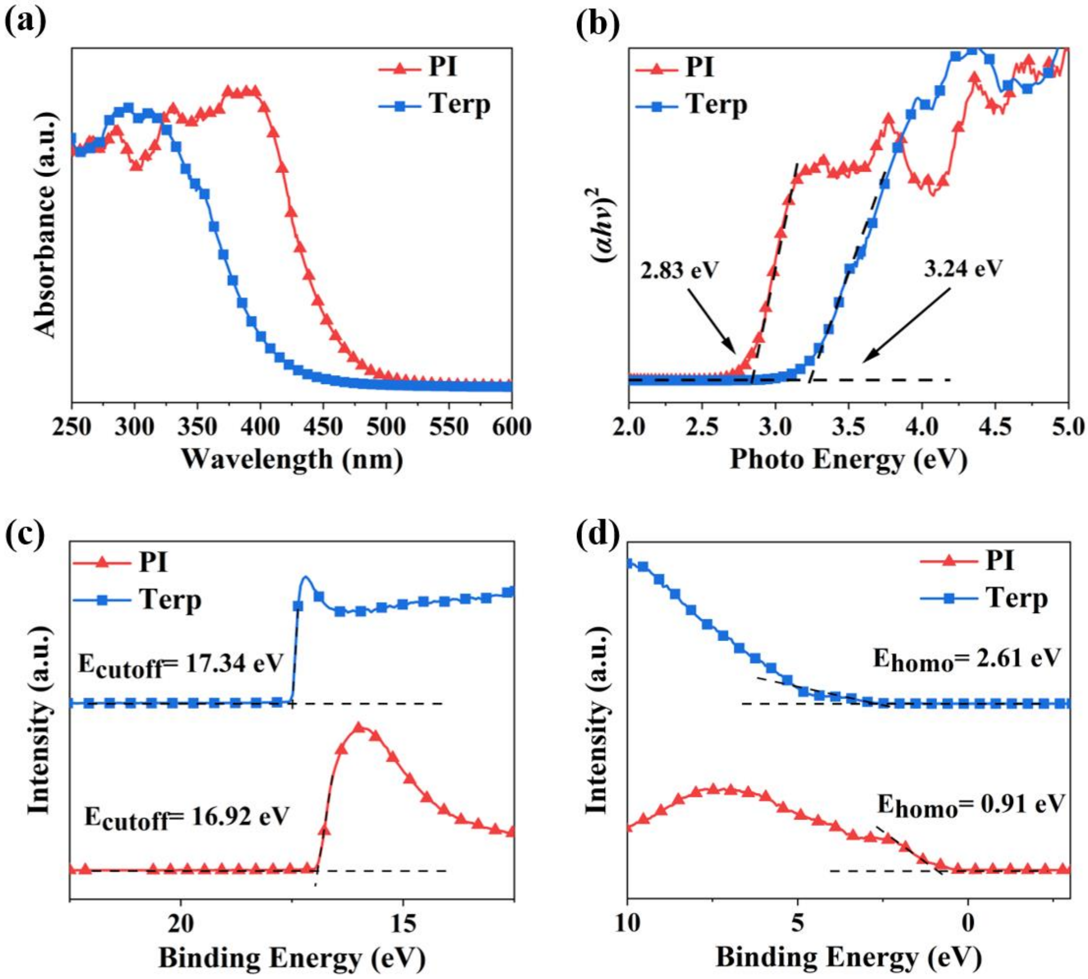

Figure S11. Energy band diagrams at the interface between terpolymer filler and PI substrate in the Terp/PI composite film.

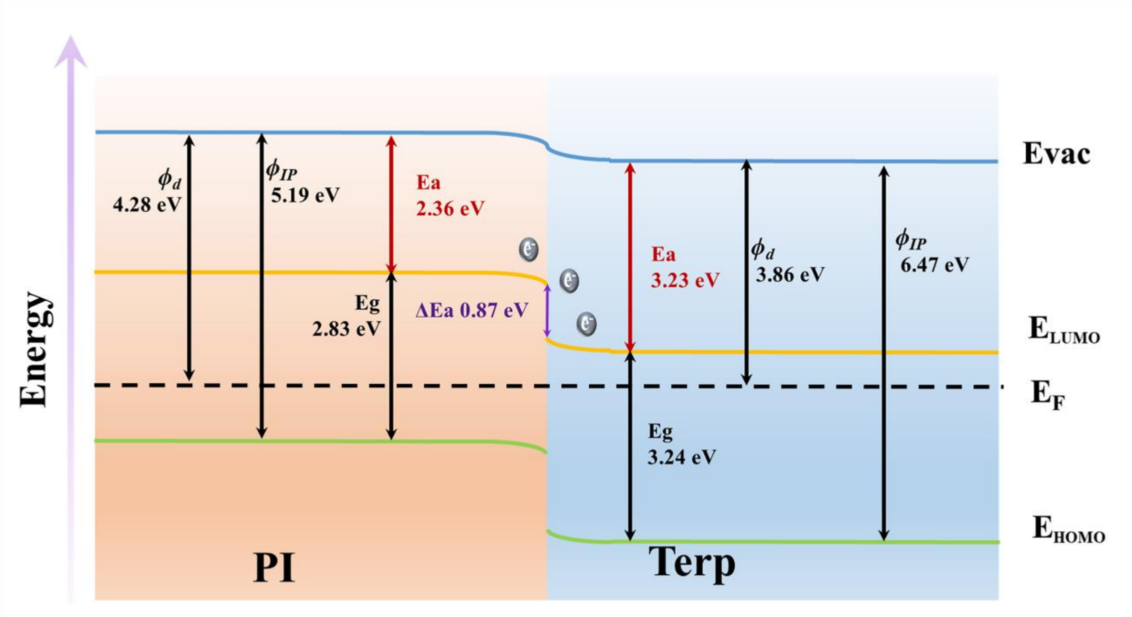

Figure S12. P-E loop of the Terp/PI-5 film after the film was heated at 150°C for (a) 30min, (b) 60 min, and (c) 90 min. (d) Calculated energy density and charge-discharge efficiency of Terp/PI-5 at 150°C.

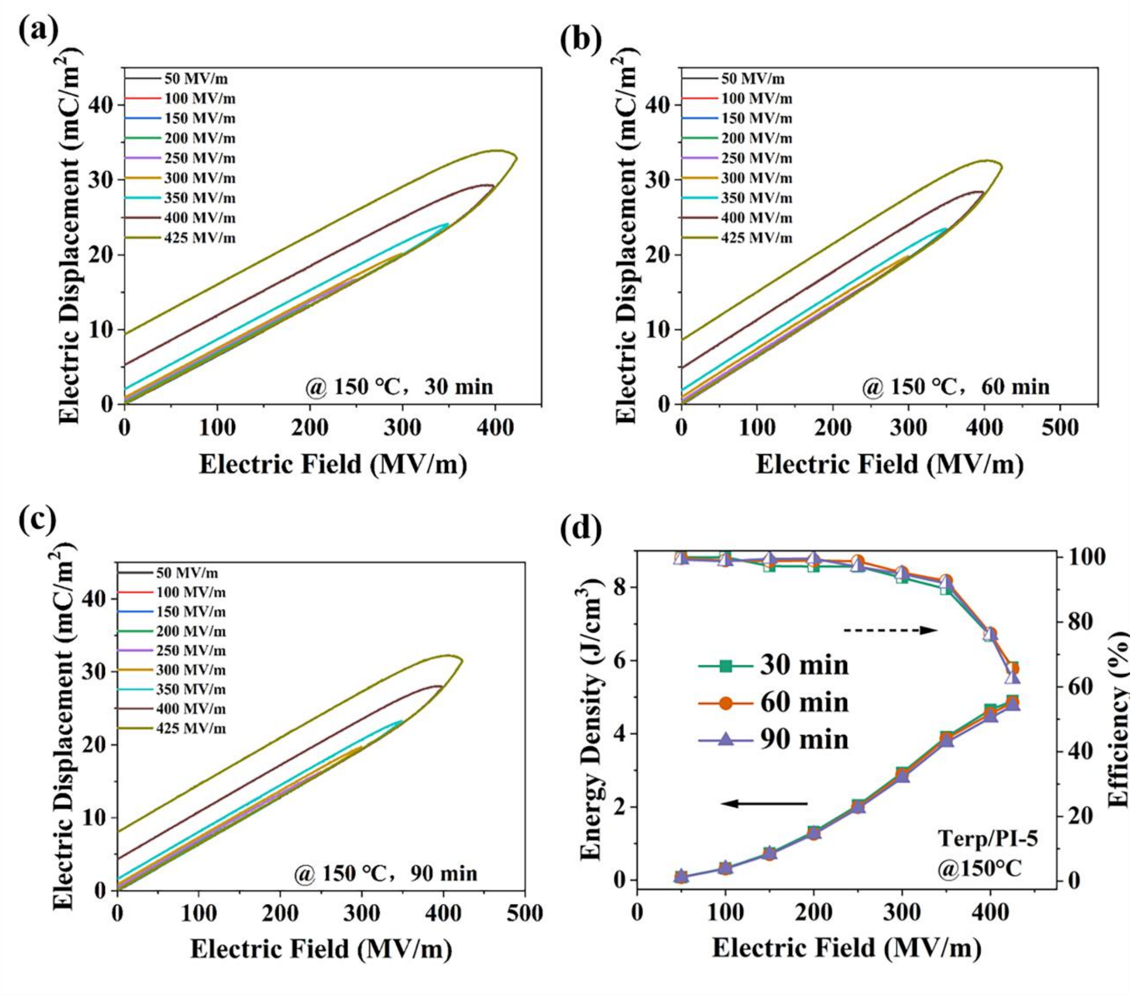

Figure S13. The scatter whiskers (error) of calculated (a) energy density and (b) charge-discharge efficiency at 25°C. The scatter whiskers (error) of calculated (c) energy density and (d) charge-discharge efficiency at 150°C.

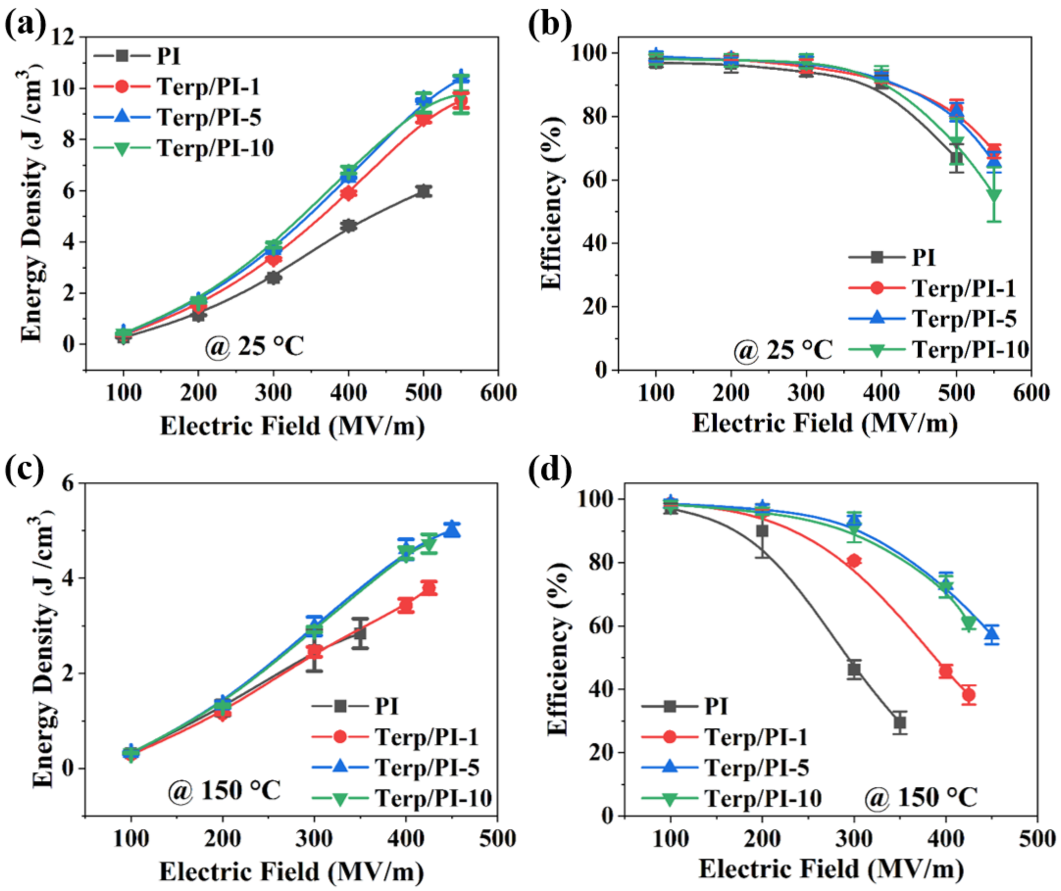

Figure S14. Bipolar P-E loop at room temperature of (a) pristine PI, (b) Terp/PI-1, (c) Terp/PI-5, (d) Terp/PI-10 films.

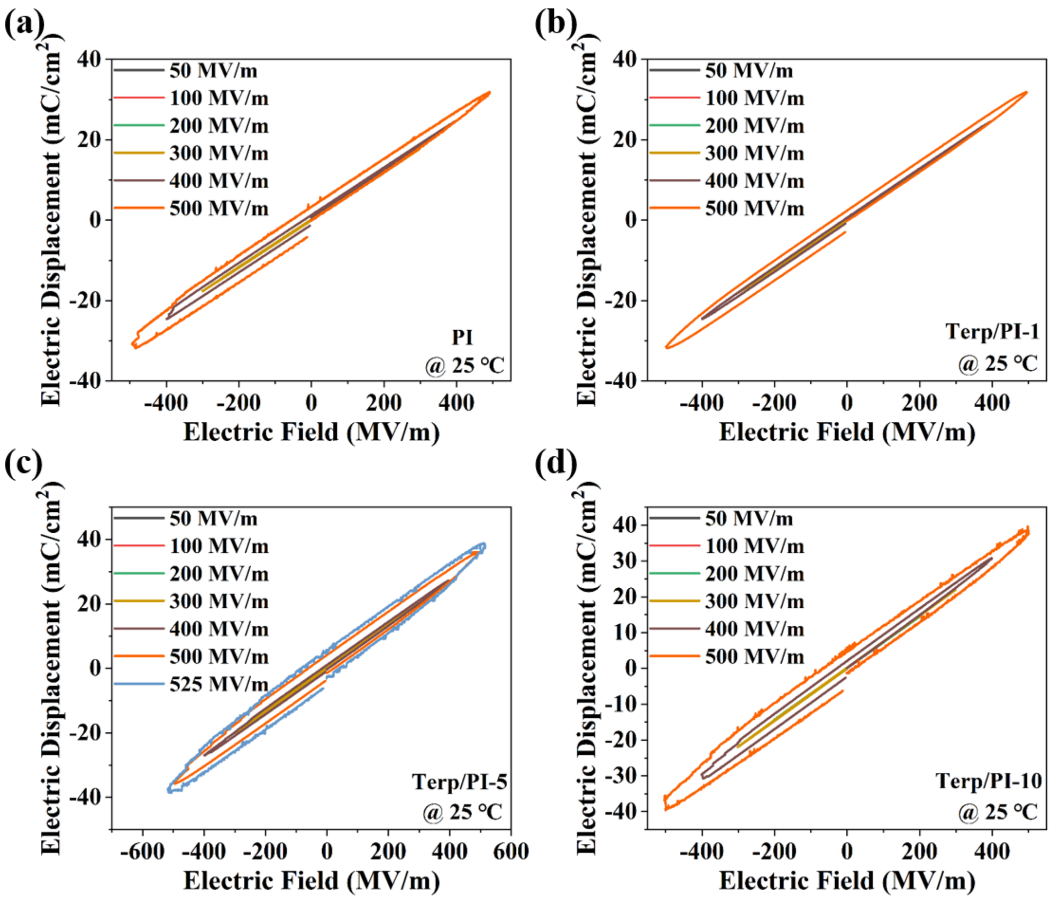

Figure S15. Bipolar P-E loop at 150 °C of (a) pristine PI, (b) Terp/PI-1, (c) Terp/PI-5, (d) Terp/PI-10 films.

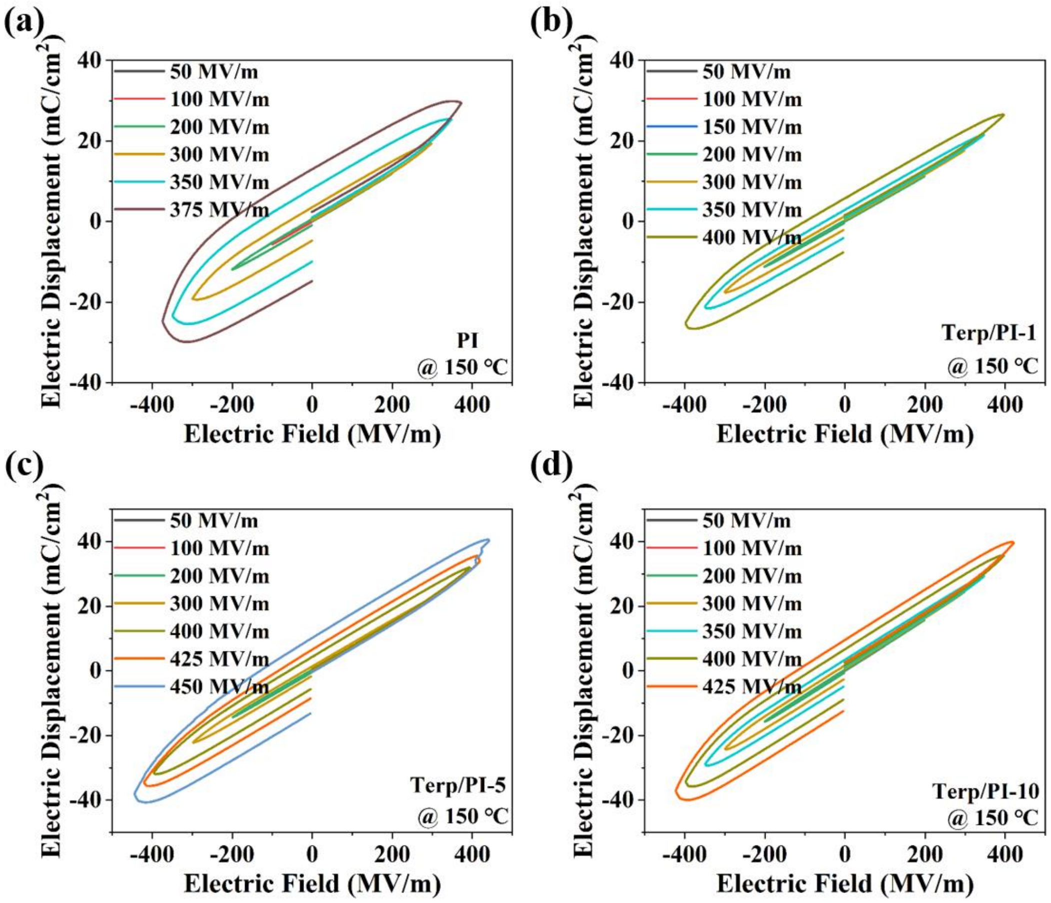

Figure S16. (a) P-E loop and (b) Weibull distribution of breakdown electric field of P(VDF-TrFE-CFE) film at room temperature.

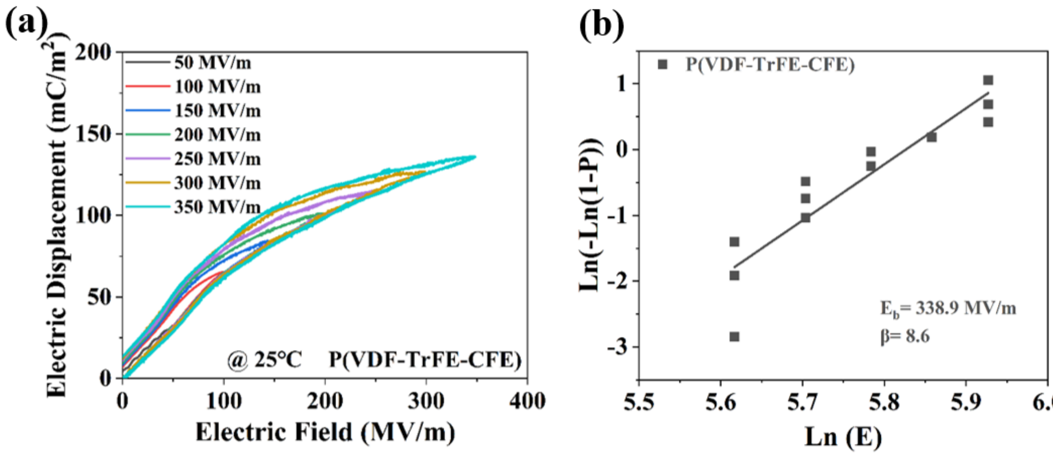

Table S1 Average chain space in Terp/PI composite films with different terpolymer contents.

Content (wt%)	2 θ	Average chain space d (Å)
PI	18.3 °	4.84
Terp/PI-1	18.6 °	4.73
Terp/PI-5	19.5 °	4.54
Terp/PI-10	20.5 °	4.32

Table S2 Young's modulus and glass transition temperature (Tg) of Terp/PI composites obtained from dynamic mechanical analysis (DMA) data.

Content (wt%)	Young's modulus (GPa)	Tg (°C)
PI	2.96	401.3
Terp/PI-1	3.28	405.7
Terp/PI-5	3.33	412.8
Terp/PI-10	2.86	404.4

Table S3 Summary of TGA data for Terp/PI composite with various terpolymer contents.

Content (wt%)	Weight loss temperature	Residue at 700℃
PI	575.4 ℃	58.85%
Terp/PI-1	573.2 ℃	59.57%
Terp/PI-5	575.8 ℃	60.62%
Terp/PI-10	561.7 ℃	59.38%

Table S4 Comparison data for the energy density for different PI-based composite at 150 °C.

Dielectric material	U_d (J cm^{-3})	Electric field (MV/m)	Reference
**Terp/PI**	5.06	475	This work
**P(VDF-HFP)/PI**	3.16	400	[1]
**BNTFs/PI**	2.98	400	[2]
**BT@PDA/PI**	1.94	315	[3]
**BZT-BCT/PI**	1.83	350	[4]
**ArTPU/PI**	4	443	[5]
**MoS_2/PI**	3.92	375	[6]
**PEI/PI/PEI**	3.2	375	[7]
**PI/PVDF/PI**	2.58	300	[8]
**MgO/PI**	4.78	433	[9]
**Al_2O_3/ PI**	2.45	400	[10]

Supplementary Note S1.

Schottky emission is a common low field conduction mechanism. Carriers overcome the energy barrier at the electrode/dielectric interface and are injected into the dielectric. As the electric field increases, the barrier height decreases due to the superposition of the image charge potential with the external potential. The Schottky barrier can be described as [11]:

$$J = AT^2 exp\ (\frac{-q(\Phi_B - \sqrt{qE/4\pi\varepsilon_r\varepsilon_0})}{k_B T}) \quad \text{(S1)}$$

$$A = \frac{4\pi q {k_B}^2 m_0}{h^3} \quad \text{(S2)}$$

where A is the effective Richardson constant (A m^{-2} K^{-2}), T is the temperature in K, e is the elementary charge, Φ_B is the interface energy barrier (eV), E is the applied electric field during current density measurement, k_B is the Boltzmann's constant, k is the dielectric constant, ε_r and ε_0 are the relative permittivity and vacuum permittivity respectively.

Equation S1 can be transformed as:

$$\ln\left(\frac{J}{T^2}\right) = \left(\frac{\sqrt{e^3/4\pi k\varepsilon_0}}{k_B T}\right)\sqrt{E} + \ln(A) - (\frac{\Phi_B}{k_B T}) \quad \text{(S3)}$$

According to equation S3, the curve of ln (J/T^2) and$\sqrt{E}$ shows a linear relationship, and the dielectric constant which is derived from the slope of the fitted curve can be used to determine whether the conduction mechanism in the dielectric is Schottky emission. The fitting results are summarized in **Table S5**, where ε_f is the dielectric constant obtained from fitting results and ε_m is the dielectric constant obtained by experimental measurement.

Table S5 The fitting results of the Schottky emission mechanism.

Content (wt%)	slope	ε_f	ε_m
PI	0.55324	3.13	3.2
Terp/PI-1	0.53671	3.33	3.47
Terp/PI-5	0.48466	4.08	3.72
Terp/PI-10	0.4625	4.49	4.24

Supplementary Note S2. Hopping conduction mechanism in higher filed.

Hopping conduction describe the trapped electrons "hopping" from one trap site to another in dielectric films with tunneling effect. The expression of hopping conduction is [12,13]:

$$J = 2q\lambda n\nu * \exp\left(-\frac{E_a}{k_B T}\right) * \sinh\left(\frac{q\lambda E}{2k_B T}\right) \quad \text{(S4)}$$

where λ is the mean hopping distance (mean spacing between trap sites), n is the electron concentration in the conduction band of the dielectric, ν is the frequency of thermal vibration of electrons at trap sites, and E_a is the activation energy, E is the electric field, q is the electron charge, k_B is the Boltzmann constant and T is the temperature, respectively. The above equation can be simplified as:

$$J = J_0 * \sinh(\alpha * E) \quad \text{(S5)}$$

where J_0 and α are two lumped parameters.

Reference

1. Feng, Q.-K.; Liu, D.-F.; Zhang, Y.-X.; Pei, J.-Y.; Zhong, S.-L.; Hu, H.-Y.; Wang, X.-J.; Dang, Z.-M. Significantly improved high-temperature charge-discharge efficiency of all-organic polyimide composites by suppressing space charges. *Nano Energy* **2022**, *99*, https://doi.org/10.1016/j.nanoen.2022.107410.
2. Hu, P.; Sun, W.; Fan, M.; Qian, J.; Jiang, J.; Dan, Z.; Lin, Y.; Nan, C.-W.; Li, M.; Shen, Y. Large energy density at high-temperature and excellent thermal stability in polyimide nanocomposite contained with small loading of BaTiO3 nanofibers. *Appl. Surf. Sci.* **2018**, *458*, 743–750, https://doi.org/10.1016/j.apsusc.2018.07.128.
3. Wu, Z.; Zhou, H.; Guo, Q.; Liu, Z.; Gong, L.; Zhang, Q.; Zhong, G.; Li, Z.; Chen, Y. Enhanced dielectric properties in polyimide nanocomposites containing barium titanate@ polydopamine core-shell nanoparticles. *J. Alloys Compd.* **2020**, *845*, 156171, https://doi.org/10.1016/j.jallcom.2020.156171.
4. Chi, Q.; Gao, Z.; Zhang, T.; Zhang, C.; Zhang, Y.; Chen, Q.; Wang, X.; Lei, Q. Excellent Energy Storage Properties with High-Temperature Stability in Sandwich-Structured Polyimide-Based Composite Films. *ACS Sustain. Chem. Eng.* **2018**, *7*, 748–757, https://doi.org/10.1021/acssuschemeng.8b04370.
5. Ahmad, A.; Tong, H.; Fan, T.; Xu, J. Binary polymer blend of ArPTU/PI with advanced comprehensive dielectric properties and ultra-high thermally stability. *J. Appl. Polym. Sci.* **2021**, *138*, 50997, https://doi.org/10.1002/app.50997.
6. Li, J.; Zhang, J.; Zhang, S.; Ren, K. 2D MoS2 nanosheet-based polyimide nanocomposite with high energy density for high temperature capacitor applications. *Macromol. Mater. Eng*. **2021**, *306*, 2100079.
7. Niu, Y.; Dong, J.; He, Y.; Xu, X.; Li, S.; Wu, K.; Wang, Q.; Wang, H. Significantly enhancing the discharge efficiency of sandwich-structured polymer dielectrics at elevated temperature by building carrier blocking interface. *Nano Energy* **2022**, *97*, https://doi.org/10.1016/j.nanoen.2022.107215.
8. Yu, S.; Liu, Y.; Ding, C.; Liu, X.; Liu, Y.; Wu, D.; Luo, H.; Chen, S. All-organic sandwich structured polymer dielectrics with polyimide and PVDF for high temperature capacitor application. *J. Energy Storage* **2023**, *62*, https://doi.org/10.1016/j.est.2023.106868.
9. Wang, P.; Guo, Y.; Zhou, D.; Li, D.; Pang, L.; Liu, W.; Su, J.; Shi, Z.; Sun, S. High-Temperature Flexible Nanocomposites with Ultra-High Energy Storage Density by Nanostructured MgO Fillers. *Adv. Funct. Mater.* **2022**, *32*, https://doi.org/10.1002/adfm.202204155.
10. Ai, D.; Li, H.; Zhou, Y.; Ren, L.; Han, Z.; Yao, B.; Zhou, W.; Zhao, L.; Xu, J.; Wang, Q. Tuning Nanofillers in In Situ Prepared Polyimide Nanocomposites for High-Temperature Capacitive Energy Storage. *Adv. Energy Mater.* **2020**, *10*, https://doi.org/10.1002/aenm.201903881.
11. Yuan, C.; Zhou, Y.; Zhu, Y.; Liang, J.; Wang, S.; Peng, S.; Li, Y.; Cheng, S.; Yang, M.; Hu, J.; et al. Polymer/molecular semiconductor all-organic composites for high-temperature dielectric energy storage. *Nat. Commun.* **2020**, *11*, 1–8, https://doi.org/10.1038/s41467-020-17760-x.
12. Yang, M.; Wang, S.; Fu, J.; Zhu, Y.; Liang, J.; Cheng, S.; Hu, S.; Hu, J.; He, J.; Li, Q. Quantum Size Effect to Induce Colossal High-Temperature Energy Storage Density and Efficiency in Polymer/Inorganic Cluster Composites. *Adv. Mater.* **2023**, *35*, e2301936, https://doi.org/10.1002/adma.202301936.
13. Yuan, C.; Liu, D.-D.; Zhu, Y.-J.; Zeng, T.; Jiang, B.-X.; Tang, C.-X.; Zhou, Y.; He, J.-L.; Li, Q. Effect of charge transport on electrical degradation in polypropylene/organic molecular semiconductor composites for HVDC cable insulation. *Appl. Phys. Lett.* **2023**, *122*, 112904, https://doi.org/10.1063/5.0133417.
